# Antibodies against *Porphyromonas gingivalis* in serum and saliva and their association with rheumatoid arthritis and periodontitis. Data from two rheumatoid arthritis cohorts in Sweden

**DOI:** 10.3389/fimmu.2023.1183194

**Published:** 2023-05-30

**Authors:** Anna Svärd, Alf Kastbom, Karin Roos Ljungberg, Barbara Potempa, Jan Potempa, G. Rutger Persson, Stefan Renvert, Johan Sanmartin Berglund, Maria K. Söderlin

**Affiliations:** ^1^ Center for Clinical Research Dalarna, Uppsala University, Uppsala, Sweden; ^2^ Department of Biomedical and Clinical Sciences, Linköping University, Linköping, Sweden; ^3^ Department of Oral Immunity and Infectious Diseases, University of Louisville School of Dentistry, Louisville, KY, United States; ^4^ Department of Microbiology, Faculty of Biochemistry, Biophysics and Biotechnology, Jagiellonian University, Krakow, Poland; ^5^ Department of Periodontics, University of Washington, Seattle, WA, United States; ^6^ Department of Oral Medicine, University of Washington, Seattle, WA, United States; ^7^ Faculty of Health Sciences, Kristianstad University, Kristianstad, Sweden; ^8^ Department of Health, Blekinge Institute of Technology, Karlskrona, Sweden; ^9^ School of Dental Science, Trinity College, Dublin, Ireland; ^10^ Faculty of Dentistry, University of Hong Kong, Hong Kong, Hong Kong SAR, China; ^11^ Department of Clinical Sciences, Section of Rheumatology, Lund University, Lund, Sweden

**Keywords:** rheumatoid arthritis, periodontitis, porphyromonas gingivalis, anti-citrullinated antibodies (ACPAs), saliva, gingipain and periodontitis

## Abstract

**Background:**

Periodontitis and oral pathogenic bacteria can contribute to the development of rheumatoid arthritis (RA). A connection between serum antibodies to *Porphyromonas gingivalis* (*P. gingivalis*) and RA has been established, but data on saliva antibodies to *P. gingivalis* in RA are lacking. We evaluated antibodies to *P. gingivalis* in serum and saliva in two Swedish RA studies as well as their association with RA, periodontitis, antibodies to citrullinated proteins (ACPA), and RA disease activity.

**Methods:**

The SARA (secretory antibodies in RA) study includes 196 patients with RA and 101 healthy controls. The Karlskrona RA study includes 132 patients with RA ≥ 61 years of age, who underwent dental examination. Serum Immunoglobulin G (IgG) and Immunoglobulin A (IgA) antibodies and saliva IgA antibodies to the *P. gingivalis*–specific Arg-specific gingipain B (RgpB) were measured in patients with RA and controls.

**Results:**

The level of saliva IgA anti-RgpB antibodies was significantly higher among patients with RA than among healthy controls in multivariate analysis adjusted for age, gender, smoking, and IgG ACPA (p = 0.022). Saliva IgA anti-RgpB antibodies were associated with RA disease activity in multivariate analysis (p = 0.036). Anti-RgpB antibodies were not associated with periodontitis or serum IgG ACPA.

**Conclusion:**

Patients with RA had higher levels of saliva IgA anti-RgpB antibodies than healthy controls. Saliva IgA anti-RgpB antibodies may be associated with RA disease activity but were not associated with periodontitis or serum IgG ACPA. Our results indicate a local production of IgA anti-RgpB in the salivary glands that is not accompanied by systemic antibody production.

## Introduction

A mucosal association to development of rheumatoid arthritis (RA) is becoming generally accepted, and the lungs as well as the intestine and the oral cavity have been implicated as possible initiating sites (1–5). Regarding the oral cavity, the periodontis-associated pathogen *Porphyromonas gingivalis* (*P. gingivalis*) is one of the mechanisms discussed. A connection between serum antibodies to *P. gingivalis* and RA has been established (6), but data on saliva antibodies to *P. gingivalis* in RA are lacking. The presence of saliva antibodies to Arg-specific gingipain B (RgpB) in RA has not been investigated previously and may provide additional clues to explain the link between inflammation of the oral mucosa and RA.

RA and periodontitis (PD) are two chronic diseases associated with elevated levels of circulating pro-inflammatory cytokines and destruction of soft tissue and bone (7, 8). *P. gingivalis*, a Gram-negative anaerobic bacterium, is a major etiological agent of PD unique among bacteria with respect to expression of peptidylarginine deiminase (PPAD), which catalyzes the posttranslational modification of arginine residues to citrulline. *P. gingivalis* has arginine-specific (RgpA and RgpB) and lysine-specific (Kgp) cysteine proteases, gingipains, expressed on the surface of the bacterial outer membrane, which are essential for attachment, colonization, and evasion of host defense (9). *P. gingivalis* has been hypothesized to play a causative role in RA by inducing the production of antibodies to citrullinated proteins (ACPA) (10), and, recently, the ability of C-terminal citrullinated peptides generated by concerted action of Arg-specific gingipains and PPAD to breach immunotolerance was implicated as a causal link (11).

A meta-analysis by Bae and Lee reported that serum *P. gingivalis* antibody levels in the RA group were higher as compared to controls, whereas, in the ACPA-positive group, serum antibody levels were significantly higher compared to that in the ACPA-negative group (12). These results confirmed the results of an earlier meta-analysis by Bender et al., which showed higher antibody levels for serum *P. gingivalis* IgG in RA as compared to systemically healthy controls with and without PD (13).

We have previously shown in the Karlskrona RA study in Sweden that PD was associated with RA with an odds ratio (OR) of 2.5 as compared to age-matched controls from the normal population from Karlskrona city (14) and that ACPA in serum and saliva were not associated with PD (15).

In the present study of antibodies to RgpB in serum and saliva, we used two well-characterized cohorts of patients with typical RA in Sweden with long-standing disease, comorbidities, systemic conditions, and anti-rheumatic medication. We had access to paired samples of serum and saliva and also clinical PD status from the Karlskrona RA study. Our hypotheses were that patients with RA would have higher levels of antibodies to RgpB than healthy controls, that anti-RgpB antibodies would be associated with higher disease activity of RA, and that patients with RA with PD would have higher levels of antibodies to RgpB than patients with RA with no PD.

## Materials and methods

### The SARA study

Patients with established RA (n = 196) and healthy controls (n = 101) were included in the SARA (secretory antibodies in RA) study in Sweden, which has been described in detail previously (16). Patients with a clinical diagnosis of RA (M05 and M06, ICD-10) and a planned follow-up visit were randomly selected for inclusion. Healthy controls were recruited among blood donors from the same geographical region. Disease activity among patients with RA was measured by the disease activity score of 28 joints using erythrocyte sedimentation rate (ESR) (DAS28ESR) (www.das-score.nl). Paired serum and saliva samples were collected from both patients with RA and healthy controls. Serum samples from 195 of the 196 patients with RA and all 101 controls, as well as saliva samples from 188 of the 196 patients with RA and 100 of the 101 controls, were available for analysis of anti-RgpB antibodies.

### The Karlskrona RA study

The Karlskrona RA study has been described in detail in previous studies (14, 15, 17). Briefly, between October 2013 and January 2015, all individuals with a clinical diagnosis of RA (M05 and M06, ICD-10) ≥ 61 years of age living in Karlskrona city in Southern Sweden were identified from the electronic regional database (Region Blekinge, Sweden). The patients with RA were invited per mail once to a rheumatological consultation with examination at the outpatient clinic at the Rheumatology Department. No data on previous antibiotic treatment or previous periodontal treatment were available. A total of 132 of the 242 (55%) patients with RA were recruited, and 83% of the patients with RA fulfilled the 1987 ACR classification criteria (18) and 72% fulfilled the 2010 ACR/EULAR classification criteria (19) for RA. A total of 89% of the patients had regular dental healthcare at least once a year. Serum samples from 130 of the 132 patients with RA and saliva samples from 111 of the 132 patients with RA were available for analysis of anti-RgpB antibodies.

### Definition of gingivitis and periodontitis (Karlskrona RA study)

A dental hygienist performed the clinical dental examinations. Panoramic radiographs were assessed by a periodontist (author RGP) masked to clinical dental and medical data. Gingivitis was defined as having ≥ 20% of measured sites with evidence of bleeding on probing. PD was defined as the clinical presence of bleeding on probing at > 20% of recorded tooth surfaces, presence of > 2 non-adjacent sites with a periodontal probing depth (PPD) ≥ 5 mm, presence of bone loss at ≥ 2 sites with a distance between the cementoenemel junction (CEJ) and bone level of ≥ 5 mm, or if evidence of a furcation invasion at molar teeth was found either clinically (grade II) or clearly visible on panoramic radiographs and bone loss ≥ 5 mm at ≥ 30%. The definition of PD was based on 2013 standards and included only individuals with chronic PD.

### Samples and antibody analyses

Saliva sampling was performed using passive secretion, with study participants drooling for 10 min into a test tube placed on ice. Patients unable to provide 0.5 ml or more of saliva were excluded from the study, which means that patients with severe Sjögren’s disease were most likely excluded. The samples were centrifuged for 5 min at 5,000 g, and the supernatant was stored at −80°C until analysis (16). Serum samples were stored at −80°C until analysis.

### RgpB antibody analyses

Anti-RgpB antibodies were analyzed by an Enzyme-linked immunosorbent assay (ELISA) developed by Quirke et al. (20) and modified by Kharlamova et al. (6). In short, the recombinant RgpB antigen expressed by genetically modified *P. gingivalis* W83 strain and purified as described earlier (21) was diluted to 2.8 µg/ml in coating buffer, and 96-well Nunc high-binding plates were coated with 100 μl per well, incubated overnight at 4°C, washed four times with PBS-Tween (0.05%), and blocked with 2% Bovine serum albumin (BSA) overnight at 4°C. Saliva was diluted 1:20, serum was diluted at 1:800 (for IgG RgpB) or 1:100 (for IgA RgpB) in a Tris-HCl buffer (pH 7.6), and 100 μl per well was added in duplicates and incubated 1.5 hours at room temperature. Plates were washed four times, and secondary antibody was added: anti-IgG (Goat Anti-Human IgG, Jackson ImmunoResearch) at a dilution of 1:10,000 or anti-IgA (Polyclonal Rabbit Anti-Human IgA/HRP, DakoCytomation) at a dilution of 1:2,000. Tetramethylbenzidine (TMB) substrate (Sigma) was added (100 μl per well), and, after 25 min, the reaction was stopped by adding 0.5M H_2_SO_4_ (100 μl per well); absorbance was measured at 450 nm. The inter-assay assay variation was 8.2% for serum IgG anti-RgpB antibodies, 7.6% for serum IgA anti-RgpB antibodies, and 9.8% for saliva IgA anti-RgpB antibodies. A serial dilution of the same high-level serum was used on each plate to create a reference curve, to obtain arbitrary units (AU).

### ACPA analyses

IgG and IgA ACPA in serum and IgA ACPA in saliva samples have been analyzed previously (16). Serum IgG antibodies to citrullinated peptides were analyzed using the second-generation anti-CCP immunoassay (Svar Life Science, Malmö, Sweden). IgA ACPA was analyzed similarly, but using an anti-human α-chain antibody as secondary antibody (22). Cutoff limit for IgG ACPA positivity was set at 25 U/ml according to the manufacturer’s instructions and for IgA ACPA at 25 AU/ml, corresponding to the 99th percentile of 101 healthy blood donors.

### Ethical approval

The Regional Ethical Review Boards at Uppsala and Lund Universities, Sweden, approved the studies (Uppsala 2011/159, LU 2013/323). All procedures performed in studies involving human participants were in accordance with the ethical standards of the institutional research committees of Uppsala and Lund University in Sweden and with the 1964 Helsinki declaration and its later amendments or comparable ethical standards. Informed consent was obtained from all individual participants included in the study.

### Statistics

Mann–Whitney U-test was used to evaluate the difference in anti-RgpB antibody levels among patients with RA versus controls and among patients with RA positive versus negative for IgG ACPA in serum. Chi-square was used for categorical variables. Spearman’s correlation coefficient was used to study the correlation of the levels of anti-RgpB antibodies and DAS28ESR as a continuous variable.

To evaluate the difference in anti-RgpB antibody levels between patients with RA and healthy controls, binary logistic regression analyses using RA as the dependent variable were performed with either saliva IgA RgpB antibodies or serum IgG or IgA RgpB antibodies as the independent variables, adjusted for age, gender, smoking status, and serum IgG ACPA status (positive or negative).

To evaluate the difference in anti-RgpB antibodies between patients with RA with and without PD, binary logistic regression analyses were performed using PD as the dependent variable and either saliva IgA antibodies to RgpB or serum IgG or IgA antibodies to RgpB as the independent variable, adjusted for age, gender, disease duration, smoking status, DAS28ESR, socioeconomic status, body mass index, and serum IgG ACPA status (positive or negative).

**Figure 1:**
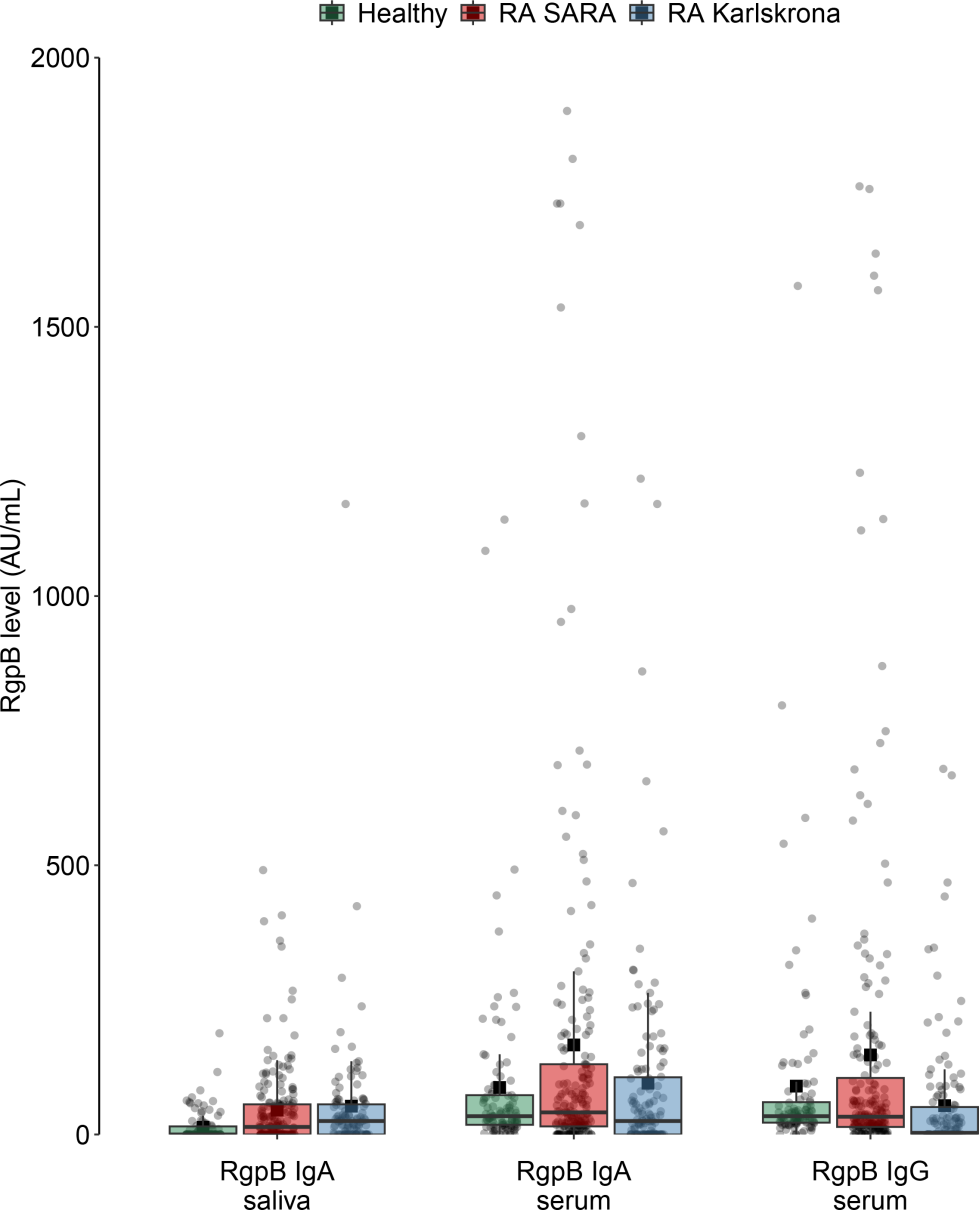
Levels of anti-RgpB antibodies among healthy controls and among patients with RA on the SARA and Karlskrona studies. All individual values are symbolized by gray dots, median value, and quartiles in the boxplots, and mean values symbolized by black squares.

To evaluate whether anti-RgpB antibodies were associated with disease activity, binary logistic regression analyses were performed using DAS28ESR remission (< 2.6) or DAS28ESR moderate/high disease activity (>3.2) versus remission/low disease activity as the dependent variable and anti-RgpB antibodies (IgG and IgA in serum and IgA in saliva) as the independent variables, adjusted for age, gender, and smoking status. Simulations in the statistical program R (version 4.2.2) were used to confirm goodness of fit of data in the logistic regression analyses in the SARA study and to create **Figure 1**. The significance level was set at α < 0.05. Statistical analyses were performed using IBM SPSS Statistics version 28.0.0.0 (Armonk, NY, USA).

## Results

**Table 1** shows the demographics, disease activity, and anti-rheumatic medication in the SARA and Karlskrona RA studies and the healthy controls in the SARA study. **Figure 1** shows the levels of the different anti-RgpB antibodies in both studies. The corresponding numbers are provided in **Supplementary Table 1**.

**Table 1:** Baseline characteristics and disease activity variables in the Karlskrona RA study and the SARA study.

Variable	Karlskrona RA study N = 132	SARA study	
		RA N = 196	Controls N = 101
Females %	71%	80%	53%
Age years, mean (SD)	70 (6.6)	64 (13.3)	49 (14.0)
Disease duration years, mean (SD)	14 (13.0) N = 127	12 (10.5) N = 193	–
Ever smokers %	62%	52%	36%
Never smoker %	38%	48%	64%
RF positive %	58% N = 129	74%	–
Serum IgG ACPA positive %	67%	80%	0%
VAS pain, mean (SD)	33 (28) N = 129	29 (23) N = 163	–
VAS global, mean (SD)	33 (26) N = 131	32 (23) N = 162	–
ESR, mean (SD)	19 (16) N = 129	19 (17) N = 194	–
CRP, mean (SD)	9.4 (8.8)	7.7 (14) N = 195	–
DAS28ESR, mean (SD)	3.0 (1.1) N = 127	2.8 (1.2) N = 158	–
csDMARDs %	67%	86%	–
On biologics %	22%	37%	–
On methotrexate %	57%	76%	–
On glucocorticoids %	47%	27%	–
With periodontitis %	61%		

BMI, body mass index; VAS, visual analogue scale; ESR, erythrocyte sedimentation rate; CRP, C-reactive protein; DAS28ESR, disease activity score (28 joints) calculated with ESR; ACPA, anti-citrullinated protein antibodies; RF, rheumatoid factor; DMARD, disease-modifying anti-rheumatic drug; csDMARDs, conventional synthetic DMARDs.

### Anti-RgpB antibodies in patients with RA versus healthy controls: the SARA study

The median level of saliva IgA anti-RgpB antibodies was higher among patients with RA (14 AU/ml) than among healthy controls (0 AU/ml) in the SARA study (p < 0.001 using Mann–Whitney U-test). In a binary logistic regression analysis adjusting for age, gender, smoking, and serum IgG ACPA, there was still a significant association between the level of IgA anti-RgpB in saliva and RA, with 1.3% increased risk for having RA per increased unit of IgA anti-RgpB [odds ratio (OR) = 1.013; 95% confidence interval (CI), 1.002–1.024; p = 0.022].

There were no statistically significant associations between serum anti-RgpB antibodies and RA in the univariate analyses or in the logistic regression analyses, regarding neither IgG anti-RgpB antibodies (OR = 1.001; 95% CI, 0.999–1.002; p = 0.368) nor IgA anti-RgpB antibodies (OR = 1.001; 95% CI, 1.000–1.003; p = 0.111).

### Anti-RgpB antibodies and ACPA

No significant associations were seen between anti-RgpB antibody levels and ACPA status, in neither serum nor saliva, using Mann–Whitney U-test. In the SARA study, patients with RA positive for IgG ACPA did not have higher levels of IgG anti-RgpB in serum (p = 0.773), IgA anti-RgpB in serum (p = 0.696), or IgA anti-RgpB in saliva (p = 0.253). In the Karlskrona RA study, the corresponding values were IgG anti-RgpB in serum (p = 0.373), IgA anti-RgpB in serum (p = 0.145), and IgA anti-RgpB in saliva (p = 0.240).

### Anti-RgpB antibodies and RA disease activity

In the SARA study, correlations between IgA anti-RgpB antibodies in both saliva and serum and disease activity parameters such as DAS28ESR, swollen, and tender joint counts were highly significant but weak in the univariate analyses, with a Spearman’s rho of 0.2 to 0.3. No significant correlations were seen between IgG anti-RgpB antibodies in serum and disease activity parameters. Saliva IgA anti-RgpB antibodies were associated with DAS28ESR (r_s_ = 0.295, p < 0.001), swollen joint count (r_s_ = 0.323, p < 0.001), tender joint count (r_s_ = 0.224, p = 0.004), ESR (r_s_ = 0.212, p = 0.004), and C-reactive protein (CRP) (r_s_ = 0.179, p = 0.014). Serum IgA anti-RgpB antibodies were associated with DAS28ESR (r_s_ = 0.228, p = 0.004), swollen joint count (r_s_ = 0.203, p = 0.008), tender joint count (r_s_ = 0.202, p = 0.009), and ESR (r_s_ = 0.145, p = 0.044). When evaluating the association between anti-RgpB antibodies and disease activity in binary logistic regression analyses using DAS28ESR remission (DAS28ESR < 2.6) (yes/no) as the dependent variable, adjusting for age, gender, and smoking, no significant association could be seen; regarding IgA anti-RgpB antibodies in saliva, the OR was 1.003 (95% CI, 0.998–1.009; p = 0.251); regarding IgA anti-RgpB antibodies in serum, the OR was 1.001 (95% CI, 1.000–1.002; p = 0.085), and regarding IgG anti-RgpB antibodies in serum, the OR was 1.001 (95% CI, 1.000–1.002; p = 0.233). In the SARA study, similar non-significant results were seen when using moderate/high disease activity (≥ 3.2) versus remission/low disease activity (<3.2) as the dependent variable. In all these regression analyses, age was the one variable showing a highly significant association to DAS28ESR.

In the Karlskrona RA study, saliva IgA anti-RgpB antibodies were associated with DAS28ESR (r_s_ = 0.232, p = 0.017), swollen joint count (r_s_ = 0.207, p = 0.03), and tender joint count (r_s_ = 0.192, p = 0.044). Serum IgG anti-RgpB antibodies were associated with CRP (r_s_ = 0.255, p = 0.003) and serum IgA anti-RgpB antibodies with CRP (r_s_ = 0.206, p = 0.019). In the binary logistic regression analyses, using DAS28ESR remission (DAS28ESR < 2.6) as the dependent variable, adjusting for age, gender, and smoking, no significant association could be seen; regarding IgA anti-RgpB antibodies in saliva, the OR was 0.994 (95% CI, 0.987–1.002; p = 0.147); regarding IgA anti-RgpB antibodies in serum, the OR was 0.998 (95% CI, 0.996–1.001; p = 0.200); and regarding IgG anti-RgpB antibodies in serum, the OR was 1.001 (95% CI, 0.997–1.004; p = 0.749). In the Karlskrona study, when using moderate/high disease activity (≥ 3.2) as the dependent variable, similar non-significant results were seen regarding serum IgG and IgA anti-RgpB antibodies. However, regarding saliva IgA anti-RgpB antibodies, a significant association was seen with an OR value of 1.008 (CI, 1.001–1.015; p = 0.036).

### Periodontitis: Karlskrona RA study

A total of 80 of the 132 (61%) patients with RA in the Karlskrona RA study had PD. There were no significant differences in level of any of the anti-RgpB antibodies between patients with and without PD (serum IgG anti-RgpB, p = 0.106; serum IgA anti-RgpB, p = 0.054; saliva IgA anti-RgpB, p = 0.086; using Mann–Whitney U-test). In the binary logistic regression analyses, neither saliva IgA anti-RgpB antibodies (OR = 0.999; 95% CI, 0.966–1.003; p = 0.703), serum IgG (OR = 1.002; 95% CI, 0.998–1.006; p = 0.306), nor serum IgA anti-RgpB antibodies (OR = 1.001; 95% CI, 0.998–1.005; p = 0.371) were significantly associated with PD.

## Discussion

This is the first study to explore saliva IgA anti-RgpB antibodies in patients with RA and healthy controls (the SARA study) and in patients with RA with and without PD (the Karlskrona RA study). The levels of saliva IgA anti-RgpB antibodies were found to be significantly higher among patients with RA than among healthy controls; among patients with RA, levels of saliva IgA anti-RgpB antibodies were associated with RA disease activity. In contrast, there was no difference in the level of serum anti-RgpB antibodies between rheumatologically healthy controls and patients with RA, and serum RgpB antibodies were not associated with RA disease activity. The other main finding was that neither serum nor saliva anti-RgpB antibodies were associated with PD among patients with RA. Methodological limitations of this study include the cross-sectional design and rather small sample size.

In our study, anti-RgpB antibodies were not associated with ACPA positivity or levels. In the meta-analysis by Bae and Lee, a positive correlation was found between *P. gingivalis* antibody levels and ACPA levels (12). However, out of the five studies in the meta-analysis (23–27), only one small study (23), including 50 patients with RA, could demonstrate a significant association between antibodies to *P. gingivalis* and IgG ACPA and one study including 78 patients with RA (24) found an association between antibodies to *P. gingivalis* and IgM ACPA, but not IgG or IgA ACPA. Kharlamova et al. (6) performed a large study including 1,974 patients with RA and found higher levels of serum IgG anti-RgpB in ACPA-positive RA compared to ACPA-negative RA, with median levels of 231 AU/ml *vs*. 166 AU/ml. Patients with RA in the study by Kharlamova et al. had a disease duration of less than 1 year, whereas our patients had a disease duration of more than 10 years. This may indicate that immunological mechanisms involving an association between ACPA and RgpB may be of importance in early RA, and possibly even before the development of arthritis, rather than in established RA. In addition, our patient cohorts were smaller.

Anti-RgpB antibody levels were not found to associate with disease activity in the regression analyses in the SARA study, whereas an association between saliva IgA anti-RgpB antibody levels and DAS28ESR could be seen in the Karlskrona study. As this association was seen only when using DAS28ESR dichotomized in moderate/high versus remission/low disease activity as the dependent variable but not when using DAS28ESR remission yes/no as the dependent variable and was not seen at all in the SARA study, the significance of this finding is unclear. It is, however, interesting to notice that it concerns saliva antibodies only and not serum antibodies. The correlation between saliva anti-RgpB antibodies and disease activity has earlier not been investigated, but correlations have been reported between serum antibodies to *P. gingivalis* and ESR (23, 25) or CRP (28). However, those studies include DMARD naïve patients (23) and patients with higher DAS28ESR, ESR, and CRP than patients in our study, most of whom are well treated with mean DAS28ESR of 2.8 and 3.0. A large majority of patients in our study are on treatment with conventional and/or biologic DMARDs. Longitudinal studies would be better suited to clarify a possible connection between antibodies to *P. gingivalis* and disease activity in RA.

In this study, serum anti-RgpB antibodies did not differ between patients with RA and controls, whereas Kharlamova et al. (6), using the same anti-RpgB method as we did, found higher levels of IgG anti-RgpB in serum in Swedish patients with RA than in non-RA controls. In our study, patients with RA had a mean disease duration of >10 years and were well treated for their RA, whereas the study by Kharlamova et al. includes patients with early RA. The difference in results may indicate that *P. gingivalis* antibodies are of importance especially in early RA.

Our results, showing a connection between saliva but not serum anti-RgpB antibodies and RA, indicate a local production of IgA anti-RgpB in the salivary glands that is not accompanied by systemic antibody production or increased prevalence of PD. This finding supports the mucosal association hypothesis, suggesting that inflammatory events in mucosal compartments, such as the oral cavity, may be an important part of RA pathogenesis. Further understanding of these events may contribute to the development of new therapeutic or even preventive strategies, possibly by reducing oral pathogens and mucosal inflammation in RA or pre-RA individuals and more active treatment of PD. However, further work is needed to delineate possible mechanisms by which mucosal immune response to *P. gingivalis* is involved in RA development or progression.

Serum anti-RgpB antibody levels were found to be lower in the Karlskrona RA study than in the SARA study. We do not have a good explanation for this. The populations are similar, although the Karlskrona RA patients are older (all being ≥ 61 years of age), and we possibly have a small sample size problem in the Karlskrona RA study.

Earlier studies on non-RA patients have shown that serum and saliva *P. gingivalis* antibodies are associated with PD (29, 30) or with PPD and clinical attachment levels (31). In the present study on patients with RA, such an association was not found. One reason for this different finding could be that we used RgpB as antigen when analyzing antibodies to *P. gingivalis* and not lipopolysaccharide as in previous studies. In addition, in the present study, patients were older, and they were well treated with anti-rheumatic medications as well as having medications for their comorbidities, which may influence antibody production. In the study by Pudakalkatti and Baheti, patients with any systemic diseases were excluded, which may have an impact on the differences in results (31). It is further not known whether individuals classified as having PD had an ongoing infection with *P. gingivalis* at the time of sampling. Although *P. gingivalis* has been identified as a key pathogen in the development of PD (32), it must be recognized that this anaerobic pathogen is part of a very complex biofilm that may include a large variety of pathogens linking periodontal infection to systemic disease (33). Recent evidence suggests that there are significant differences in the composition of subgingival microbiota between individuals with or without a diagnosis of PD that may also include other pathogens than *P. gingivalis* (34).

Our RA patient cohorts are representative of general Swedish RA patient populations. In addition, the Karlskrona RA study is a truly population-based cohort with systematic sampling, and, for these patients, we also had a clinical PD status. We included patients with typical RA with comorbid and systemic conditions and smokers, as well as healthy controls. Our RA patient populations were well-treated and stable. It should be recognized that all study participants had access to government-subsidized dental and medical care. The lack of association between anti-RgpB antibodies and disease activity or PD in this study may partly be explained by this, as all anti-inflammatory medication theoretically could inhibit antibody production.

It may seem counterintuitive that anti-RgpB antibodies were associated with RA but not with PD, because *P. gingivalis* is considered a major cause of PD. A possible explanation for this could be that not all individuals with *P. gingivalis* infection and PD develop antibodies to the bacterium and that this tendency to produce antibodies is associated with the RA disease. This would correspond to the specific disposition of patients with RA do develop ACPA, which non-RA individuals rarely do. In addition, the small sample size in the Karlskrona RA study may be an explaining factor.

One weakness of this study is that we did not have a clinical PD status for the SARA RA patients and healthy controls. Previous studies on the association between RA and *P. gingivalis* antibodies use different in-house ELISA techniques using different antigenic parts of the bacterium. As there is no standardized unit, direct comparisons of these studies are difficult. It is currently not confirmed whether the association of *P. gingivalis* and RA is a causal one or non-causal based on genetic risk factors and environmental factors such as smoking. Our cross-sectional study design does not allow us to assess causality.

In conclusion, the levels of saliva IgA anti-RgpB antibodies, but not serum anti-RgpB antibodies, were significantly higher among patients with RA than among healthy controls. In patients with RA, saliva IgA anti-RgpB antibodies were associated with RA disease activity in the Karlskrona study. None of the anti-RgpB antibodies were associated with PD. Anti-RgpB-antibodies were not associated with serum IgG ACPA.

## Data availability statement

The raw data supporting the conclusions of this article will be made available by the authors, without undue reservation.

## Ethics statement

The studies involving human participants were reviewed and approved by Uppsala 2011/159 and LU 2013/323. The patients/participants provided their written informed consent to participate in this study. Written informed consent was obtained from the individual(s) for the publication of any potentially identifiable images or data included in this article.

## Author contributions

AS, KL, SR, GP, JB and MS contributed to the conception and design of the study and to acquisition of data. BP and JP produced and provided RgpB antigen. AS, AK, SR, GP, JB and MS contributed to analysis and interpretation of data. AS wrote the first draft of the manuscript. MS, AK, BP and JP wrote sections of the manuscript. All authors contributed to manuscript revision and read and approved the submitted version.
