# Supplementary material for: Antibodies against Porphyromonas gingivalis in serum and saliva and their association with rheumatoid arthritis and periodontitis. Data from two rheumatoid arthritis cohorts in Sweden
Source: Front Immunol. 2023 May 30;14:1183194. doi: 10.3389/fimmu.2023.1183194 (PMC10265683; doi:10.3389/fimmu.2023.1183194)
Supplement: Supplementary file 1 [file Table_1.docx]

Supplementary Table 1. Levels of RgpB antibodies in the two RA studies.

|  | **The Karlskrona RA study**  **N=132** | | | | **The SARA study** | | | | | | | | | |
| --- | --- | --- | --- | --- | --- | --- | --- | --- | --- | --- | --- | --- | --- | --- |
|  |  |  |  |  | **RA**  **N=196** | | | | | **Controls**  **N=101** | | | | |
| **Antibody** | **Number** | **Mean (SD)** | **Median (25^th^ -75^th^ percentile)** | **Range** | **Number** | **Mean (SD)** | **Median (25^th^ -75^th^ percentile)** | **Range** | **Number** | | **Mean (SD)** | **Median (25^th^ -75^th^ percentile)** | **Range** |  |
| IgG RgpB antibodies, serum (AU/mL) | 130 | 54 (115) | 3  (0.0-53) | 0-679 | 195 | 148  (317) | 33  (14-110) | 0-1761 | 101 | | 90  (194) | 34  (22-62) | 0-1575 |  |
| IgA RgpB antibodies, serum (AU/mL) | 130 | 95 (191) | 25.0  (0-110) | 0-1218 | 195 | 166  (345) | 41  (15-131) | 0-1907 | 101 | | 87  (172) | 34  (17-74) | 0-1142 |  |
| IgA RgpB antibodies, saliva (AU/mL) | 111 | 53 (124) | 25  (0-57) | 0-1171 | 188 | 45  (78) | 14  (0-56) | 0-491 | 100 | | 13  (28) | 0  (0-15) | 0-188 |  |
